# The impact of dependent coverage expansion under the Affordable Care Act on time to breast cancer treatment among young women

**DOI:** 10.1371/journal.pone.0198771

**Published:** 2018-06-13

**Authors:** Xuesong Han, Jingxuan Zhao, Kathryn J. Ruddy, Chun Chieh Lin, Helmneh M. Sineshaw, Ahmedin Jemal

**Affiliations:** 1 Surveillance and Health Services Research, American Cancer Society, Atlanta, GA, United States of America; 2 Rollins School of Public Health, Emory University, Atlanta, GA, United States of America; 3 Department of Oncology, Mayo Clinic, Rochester, MN, United States of America; University of South Alabama Mitchell Cancer Institute, UNITED STATES

## Abstract

**Introduction:**

Breast cancer in young women tends to be more aggressive, but timely treatment may not be always available, particularly to those without health insurance. We aim to examine whether the dependent coverage expansion under the Affordable Care Act (ACA-DCE) implemented in 2010 was associated with changes in time to treatment among women diagnosed with early stage breast cancer.

**Methods:**

A total of 7,176 patients diagnosed with early stage breast cancer in 2007–2009 (pre-ACA) and 2011–2013 (post-ACA) were identified from the National Cancer Database. A quasi-experimental design difference-in-differences (DD) approach was used, with patients aged 19–25 (targeted by the policy) considered as the intervention group, and patients aged 26–34 years (not affected by the policy) as the control group. Changes in the following treatment outcomes were examined: time from diagnosis to surgery, time from surgery to adjuvant chemotherapy, and time from adjuvant chemotherapy to radiation.

**Results:**

Compared with the control group of patients aged 26–34, young patients aged 19–25 experienced a statistically nonsignificant decrease of 2.7 percentage points (95% CI [-1.2, 6.5]) in the uninsured rate. This did not translate into more reduction in delays to surgery (DD = 2.7 days, 95% CI [-3.2, 8.3]), chemotherapy (DD = -1.0 days, 95% CI [-7.2, 5.2]) or radiation (DD = 5.3 days, 95% CI [-15.6, 26.3]) in the younger cohort than the older cohort.

**Conclusions and Relevance:**

No significant changes in time to treatment were found among young women diagnosed with early stage breast cancer after the implementation of the ACA-DCE. Future studies examining impacts of health care policy reform on breast cancer care are warranted to include patients from low-income families and to consider effects from Medicaid expansion.

## Introduction

Although breast cancer rarely occurs at young age, it still is one of the most common cancers among young adults [1, 2]. Moreover, breast cancer diagnosed in women younger than 35 years tends to be more aggressive and carries a worse prognosis than in older adults [3]. While timely treatment is essential for optimized prognosis and survival of breast cancer, it is not always available to patients without adequate health insurance [4–6]. This may be particularly problematic for young adults, who historically had the highest uninsured rate in the US [7]. In September 2010, the dependent coverage expansion under the Affordable Care Act (ACA-DCE) went into effect, allowing young adults to be covered under their parents’ health plans until they turn 26 years old. ACA-DCE has increased insurance coverage among the target population of young adults aged 19–25 years [8], as well as among newly diagnosed cancer patients of that age [2, 9]. However, the impact of this policy on access to breast cancer treatment among young women is unknown. This study aimed to examine if there is any change in time to treatment after the implementation of the ACA-DCE among young women diagnosed with early stage breast cancer.

## Methods

### Patients

We used data from the National Cancer Database (NCDB), a nationwide hospital-based cancer registry jointly sponsored by the American Cancer Society and the American College of Surgeons, including approximately 70% of all newly diagnosed cancer cases in the U.S.[10] From the NCDB, we identified female patients aged 19–34 years old at the time of diagnosis with a first primary stage I, IIA, IIB or IIIA-T3N1M0 breast cancer in 2007–2009 and in 2011–2013. The year 2010 was excluded as a washout/phase-in period. A quasi-experimental design difference-in-differences (DD) approach was used, with patients aged 19–25 (targeted by the policy) considered as the “intervention” group, and patients aged 26–34 years (not affected by the policy) as the “control” group. Only early stage breast cancer patients were included in the study to focus on patients who received breast surgery as their first treatment, sometimes followed by adjuvant radiation and/or systemic therapy.

**Fig 1:**
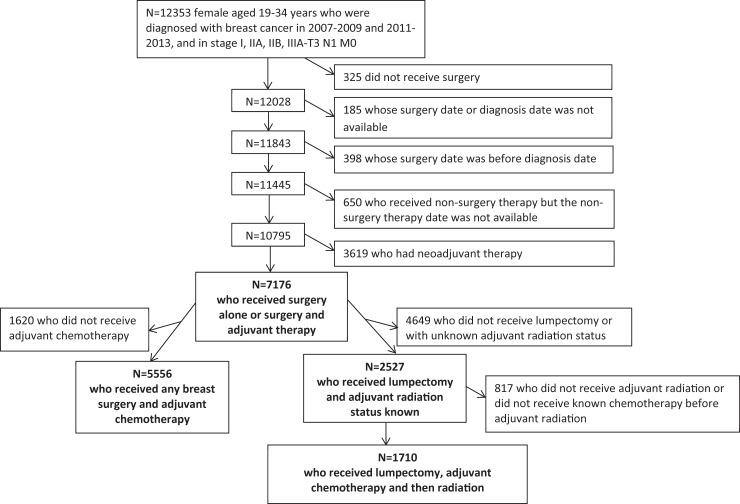
Inclusion/Exclusion diagram. The research was approved by the Morehouse University Institutional Review Board.

We excluded patients receiving no surgery, with autopsy pathology only, with local tumor destruction, or with surgery data missing (n = 325); any patient whose surgery date or diagnosis date was missing (n = 185), or with a diagnosis date after the surgery date (n = 398); patients whose radiation or systematic therapy date was missing if they received radiation or systematic therapy (n = 650); and patients who received neoadjuvant therapy (n = 3,619). Finally, a total of 7,176 female breast cancer patients were available for the analyses. Fig 1 provides a detailed inclusion/exclusion diagram.

### Outcomes

Our outcomes of interest include insurance coverage, time from diagnosis to the most definitive breast surgery, time from surgery to chemotherapy if adjuvant chemotherapy was received (N = 5556), receipt of adjuvant radiation therapy if lumpectomy was the definitive surgery (N = 2527), and time from adjuvant chemotherapy to radiation if lumpectomy and both adjuvant treatments were received (N = 1710). Those who received mastectomy were excluded in the radiation analyses because changing plastic surgery practices may have impacted the time to radiation substantially. Because previous research showed that a delay of more than 2 months from diagnosis to initial treatment [11] and a delay of 2–3 months in adjuvant therapy [5, 12–14] were associated with worse outcomes among breast cancer patients, we examined the proportion of patients who received surgery more than 2 months after the diagnosis and proportions of patients who received adjuvant therapy more than 2 months and 3 months.

### Statistical analysis

We used a difference-in-difference (DD) approach to evaluate the impact of the ACA-DCE on insurance coverage and treatment, where changes from before to after the ACA-DCE (2007–2009 vs. 2011–2013) were calculated for the intervention group of patients aged 19–25 and for the control group of patients aged 26–34. Crude uninsured rate was calculated. DD estimates and p-values for treatment outcomes were calculated using multivariable linear probability models adjusted for age, race/ethnicity, zip-code level education (percentage of residents in patient’s zip code without a high school diploma), region, stage, comorbidity score [15], and facility type. Surgery type (lumpectomy or mastectomy) and reconstruction status were also controlled in the analyses of time to surgery and time to adjuvant chemotherapy. All analyses were conducted using SAS 9.4 (SAS Institute, Cary NC). Significance level was set at 0.05, and all statistical tests were two-sided.

## Results

The study sample was composed of 6.0% patients in the intervention group and 94.0% in the control group. The majority of patients were non-Hispanic white (61.8%), privately-insured (78.4%), diagnosed at stage II (59.7%), and without comorbidity at the time of diagnosis (94.1%) (Table 1). After 2010, the uninsured rate decreased from 7.7% to 5.0% among patients aged 19–25 years, and unchanged at 4% among those aged 26–34 years, resulting a nonsignificant net decrease of 2.7 percentage points (ppt) (95% CI [-1.2, 6.5], *P* = 0.1749) among patients aged 19–25 years compared to those patients aged 26–34 years.

**Table 1:** Characteristics for young breast cancer patients, NCDB 2007–2013.

	Patients who received any breast surgery (N = 7176)	Patients who received surgery and adjuvant chemotherapy (N = 5556)	Patients who received lumpectomy (N = 2527)	Patients who received lumpectomy, adjuvant chemotherapy and radiation (N = 1710)
Year of diagnosis				
2007–2009	3466 (48.3)	2717 (48.9)	1402 (55.5)	951 (55.6)
2011–2013	3710 (51.7)	2839 (51.1)	1125 (44.5)	759 (44.4)
Age group				
19–25 years	431 (6.0)	312 (5.6)	158 (6.3)	99 (5.8)
26–34 years	6745 (94.0)	5244 (94.4)	2369 (93.7)	1611 (94.2)
Race				
Non-Hispanic white	4433 (61.8)	3453 (62.1)	1436 (56.8)	984 (57.5)
Non-Hispanic black	1204 (16.8)	961 (17.3)	512 (20.3)	364 (21.3)
Hispanic	620 (8.6)	468 (8.4)	223 (8.8)	138 (8.1)
Non-Hispanic Other	569 (7.9)	393 (7.1)	229 (9.1)	137 (8.0)
Unknown	350 (4.9)	281 (5.1)	127 (5.0)	87 (5.1)
Percentage of zip-code residents with no high school diploma				
<14%	2731 (38.1)	2099 (37.8)	951 (37.6)	643 (37.6)
14–19.9%	1544 (21.5)	1195 (21.5)	541 (21.4)	371 (21.7)
20–28.9%	1558 (21.7)	1229 (22.1)	546 (21.6)	379 (22.2
29% +	1075 (15.0)	821 (14.8)	399 (15.8)	261 (15.3)
Unknown	268 (3.7)	212 (3.8)	90 (3.6)	56 (3.3)
Primary payer				
Uninsured	293 (4.1)	217 (3.9)	118 (4.7)	77 (4.5)
Medicaid	1015 (14.1)	833 (15.0)	359 (14.2)	238 (13.9)
Medicare	117 (1.6)	87 (1.6)	49 (1.9)	31 (1.8)
Government	19 (0.3)	11 (0.2)	4 (0.2)	2 (0.1)
Private	5628 (78.4)	4341 (78.1)	1960 (77.6)	1344 (78.6)
Unknown	104 (1.5)	67 (1.2)	37 (1.5)	18 (1.1)
Region				
South	2722 (37.9)	2125 (38.2)	882 (34.9)	611 (35.7)
Northeast	1499 (20.9)	1129 (20.3)	571 (22.6)	394 (23.0)
Midwest	1801 (25.1)	1458 (26.2)	632 (25.0)	449 (26.3)
West	1118 (15.6)	821 (14.8)	429 (17.0)	249 (14.6)
Unknown	36 (0.5)	23 (0.4)	13 (0.5)	5 (1.2)
Comorbidity score*				
0	6754 (94.1)	5227 (94.1)	2402 (95.1)	1623 (94.9)
1 +	422 (5.9)	329 (5.9)	125 (4.9)	87 (5.1)
Stage				
I	2691 (37.5)	1659 (29.9)	1099 (43.5)	614 (35.9)
II	4282 (59.7)	3713 (66.8)	1402 (55.5)	1075 (62.9)
III	203 (2.8)	184 (3.3)	26 (1.0)	21 (1.2)
Facility type				
Community cancer program	599 (8.4)	457 (8.2)	253 (10.0)	171 (10.0)
Comprehensive community cancer program	2993 (41.7)	2335 (42.0)	1102 (43.6)	751 (43.9)
Teach/research	1740 (24.2)	1341 (24.1)	551 (21.8)	374 (20.6)
NCI program/network	1022 (14.2)	774 (13.9)	352 (13.9)	220 (12.9)
Community network programs	801 (11.2)	631 (11.4)	257 (10.2)	185 (10.8)
Other & unknown	21 (0.3)	18 (0.3)	12 (0.5)	9 (0.5)

Numbers in the table are: sample N (%).

* Modified weighted Charlson Deyo Score with cancer excluded from the construction of the score.

Between 2007–2009 and 2011–2013, the time from diagnosis to the most definitive breast surgery increased by several days both in patients aged 19–26 years and in those aged 26–34 years, with no significant difference in the change between the two groups (DD = 2.6 days, 95% CI [-3.2, 8.3], *P* = 0.3785) (Table 2). Similarly, we did not find a difference between the age groups in the change of the proportion of patients who received surgery later than 2 months after diagnosis (DD = -0.5 ppt, 95% CI [-7.2, 6.1], *P* = 0.8773). Similarly, among patients who received adjuvant chemotherapy, the time to chemotherapy after definitive surgery did not change in either group, with no difference in the change between the groups (DD = -1.0 days, 95% CI [-7.2, 5.2], *P* = 0.7530). Among patients who received lumpectomy, no significant difference in the change was observed in receipt of adjuvant radiation therapy (DD = -3.2 ppt, 95% CI [-14.6, 8.2], *P* = 0.5835) nor in time from adjuvant chemotherapy to radiation therapy (DD = 5.3 days, 95% CI [-15.6, 26.3], *P* = 0.6173) between the groups.

**Table 2:** Difference-in-differences analysis for receipt and time to treatment among young breast cancer patients, NCDB 2007–2013.

	19–25 years			26–34 years				
Treatment outcome	2007–09	2011–13	Difference and CI	2007–09	2011–13	Difference and CI	DD and CI	*P*-value
For patients who received any breast surgery (N = 7176) *								
N	209	222		3257	3488			
Days from diagnosis to the most definitive breast surgery	41.7	46.7	5.0 (-0.5, 10.6)	42.2	44.6	2.5 (1.0, 3.9)	2.6 (-3.2, 8.3)	0.3785
> 60 days in receiving the most definitive surgery (%)	22.0	22.7	0.7 (-5.7, 7.1)	20.6	21.8	1.2 (-0.4, 2.9)	-0.5 (-7.1, 6.1)	0.8773
For patients who received any breast surgery and adjuvant chemotherapy* (N = 5556)								
N	156	156		2561	2683			
Days from breast surgery to adjuvant chemotherapy	46.1	45.9	-0.2 (-6.3, 5.9)	46.1	46.9	0.8 (-0.7, 2.3)	-1.0 (-7.2, 5.2)	0.7530
> 60 days in receiving adjuvant chemotherapy (%)	19.0	19.8	0.8 (-7.0, 8.6)	21.9	22.2	0.3 (-1.6, 2.3)	0.5 (-7.5, 8.4)	0.9081
> 90 days in receiving adjuvant chemotherapy (%)	4.8	5.2	0.4 (-3.8, 4.7)	6.0	4.7	-1.3 (-2.4, -0.3)	1.8 (-2.6, 6.1)	0.4272
For patients who received lumpectomy (breast conserving surgery) ^#^ (N = 2527)								
N	90	68		1312	1057			
Receipt adjuvant radiation (%)	89.2	88.2	-1.0 (-12.0, 10.0)	87.2	89.4	2.2 (-0.7, 5.0)	-3.2 (-14.6, 8.2)	0.5835
For patients who received lumpectomy, adjuvant chemotherapy and radiation ^#^ (N = 1710)								
N	59	40		892	719			
Days from adjuvant chemotherapy to radiation	144.2	153.3	9.0 (-11.3, 29.4)	138.7	142.4	3.7 (-1.3, 8.7)	5.34 (-15.6, 26.3)	0.6173
> 60 days in receiving radiation (%)	95.6	95.9	0.3 (-5.9, 6.5)	92.7	94.5	1.8 (0.3, 3.4)	-1.5 (-7.9, 4.8)	0.6376
> 90 days in receiving radiation (%)	91.2	95.3	4.1 (-8.8, 17.0)	81.2	86.6	5.4 (2.3, 8.6)	-1.3 (-14.6, 11.9)	0.8438

CI = 95% confidence interval; DD = difference-in-differences. P-values are for DD.

* Models were adjusted for surgery type (lumpectomy or mastectomy), reconstruction status, age, race/ethnicity, education, region, stage, comorbidity score and facility type.

^#^ Models were adjusted for age, race/ethnicity, zip-code level education, region, stage, comorbidity score and facility type.

## Discussion

We examined changes in insurance coverage and receipt of treatment among young women diagnosed with early stage breast cancer following the ACA dependent expansion insurance coverage using the NCDB from 2007–2013. We found a nonsignificant net decrease of 2.7 ppt in uninsured rate among patients aged 19–25 years relative to those patients aged 26–34 year following the ACA, which was comparable to the findings of two previous studies on young adult cancer patients using population-based cancer registry data [2, 9], where a net decrease in uninsured rate of 3.1 ppt and 2.0 ppt rate were found respectively. We did not find any significant differences in either age group or between the age groups in pre- to post- ACA changes in receipt of treatment or treatment delays including time from diagnosis to surgery, time from surgery to adjuvant chemotherapy, receipt of adjuvant radiation after lumpectomy, and time from adjuvant chemotherapy to radiation.

Although NCDB captures 70% of new cancer cases nationwide each year, breast cancer is rare among young adults, especially among the ACA-DCE extended parental insurance eligibility-targeted population of individuals with age 19–25 years old. Thus, a relatively small sample size limits the conclusiveness of our results. Also, the majority of beneficiaries of the extended parental insurance eligibility clause in the ACA-DCE, whose parents are covered by employer-sponsored or self-purchased private insurance, were likely from families that were relatively well-off financially [16]. For such patients, insurance coverage may not be the major barrier to access to treatment. Instead, increased use of different imaging modalities and delays introduced by genetic testing and frequent second opinions [17] might be more common sources of delay in socioeconomically advantaged patients. Young patients from low-income families may likely have benefited more from Medicaid expansion, another component of the ACA that was implemented in 2014 in the states that opted to expand Medicaid eligibility. Future studies exploring time to breast cancer treatment among young adults should consider the impact of Medicaid expansion along with the ACA-DCE.

Limitations of our study include relatively short follow-up time since the ACA-DCE, which is especially important given that there is inevitably a time lag between a policy implementation and ultimate impact on care; limited generalizability given that our sample was from Commission-on-Cancer accredited hospitals instead of a population-based sample; lack of information on other factors that may have impacted treatment delays over this period, such as increasing adoption of genomic assays and Oncotype Dx test; and unavailability of parents’ socioeconomic status to control for in the analyses.

## Conclusions

In summary, this study found no statistically significant changes in time to breast cancer treatment among women 19–25 years old compared to slightly older women after the implementation of the ACA-DCE. Moving forward, studies examining the impact of the ACA on breast cancer care are warranted to include more patients from low-income families and to take Medicaid expansion into account.
